# Prenatal Factors Associated with Maternal Cardiometabolic Risk Markers during Pregnancy: The ECLIPSES Study

**DOI:** 10.3390/nu15051135

**Published:** 2023-02-23

**Authors:** Ehsan Motevalizadeh, Andrés Díaz-López, Francisco Martín-Luján, Josep Basora, Victoria Arija

**Affiliations:** 1 Nutrition and Mental Health Research Group (NUTRISAM), Faculty of Medicine and Health Sciences, Universitat Rovira i Virgili (URV), 43201 Reus, Spain; 2 Institut d’Investigació Sanitària Pere Virgili (IISPV), 43005 Tarragona, Spain; 3 Institut d’Investigació en Atenció Primària IDIAP Jordi Gol, Institut Català de la Salut (ICS), 08007 Barcelona, Spain; 4 Collaborative Group on Lifestyles, Nutrition, and Tobacco (CENIT), Institut d´Investigació en Atenció Primària IDIAP Jordi Gol, Institut Català de la Salut (ICS), 43202 Reus, Spain

**Keywords:** cardiometabolic risk, pregnancy, HOMA-IR, gestational weight gain, ECLIPSES

## Abstract

To examine the associations of sociodemographic, lifestyle, and clinical factors with cardiometabolic risk and each of its components during pregnancy in a pregnant population from Catalonia (Spain). A prospective cohort study of 265 healthy pregnant women (39 ± 5 years) in the first and third-trimesters. Sociodemographic, obstetric, anthropometric, lifestyle and dietary variables were collected, and blood samples were taken. The following cardiometabolic risk markers were evaluated: BMI, blood pressure, glucose, insulin, HOMA-IR, triglycerides, LDL, and HDL-cholesterol. From these, a cluster cardiometabolic risk (CCR)-z score was created by summating all z-scores (except insulin and DBP) computed for each risk factor. Data were analyzed using bivariate analysis and multivariable linear regression. In the multivariable models, the first-trimester CCRs was positively associated with overweight/obesity status (β: 3.54, 95%CI: 2.73, 4.36) but inversely related to the level of education (β: −1.04, 95%CI: −1.94, 0.14) and physical activity (PA) (β: −1.21, 95%CI: −2.24, −0.17). The association between overweight/obesity and CCR (β:1.91, 95%CI: 1.01, 2.82) persisted into the third-trimester, whereas insufficient GWG (β: −1.14, 95%CI: −1.98, −0.30) and higher social class (β: −2.28, 95%CI: −3.42, −1.13) were significantly associated with a lower CCRs. Starting pregnancy with normal weight, higher socioeconomic and educational levels, being a non-smoker, non-consumer of alcohol, and PA were protective factors against cardiovascular risk during pregnancy.

## 1. Introduction

Significant metabolic and physiological changes sustain a typical pregnancy and promote fetal growth and development [1]. However, inadequate adaptation to these changes (e.g., interrelated cardiometabolic alterations such as maternal obesity, elevated fasting glucose, insulin resistance and/or hyperinsulinemia, dyslipidemia, and elevated blood pressure (BP)) sometimes leads to serious complications that affect the health of both mother and child. It is therefore critically important to study cardiometabolic risks in pregnant women since several maternal sociodemographic and lifestyle-related risk factors can negatively influence the cardiometabolic status of pregnant women [2,3,4].

Regarding lifestyle, maternal diet quality is a potentially modifiable behavior involved in the etiology of cardiometabolic disorders during gestation [5,6,7,8,9]. Reinforcing this evidence, epidemiologic studies have reported that dietary approaches to prevent hypertension, such as a healthy diet comprising a high intake of fruit, vegetables, whole grains, and low-fat dairy products produced beneficial effects on glucose, lipid profile, and BP during pregnancy [6,8]. For example, a Mediterranean-style diet (MedDiet) has been associated with lower prenatal maternal BP [7] and cardiometabolic risk among pregnant women [5].

Evidence also suggests that a lack of physical activity (PA) from the first-trimester increases the risk of pregnancy complications (e.g., gestational hypertension, gestational diabetes mellitus (GDM), pre-eclampsia, and excessive gestational weight gain (GWG) [10]. Another well-established risk factor is smoking during pregnancy. Several studies have also linked prenatal maternal smoking to multiple adverse health outcomes for both mother [11] and child [12]. However, the role of maternal smoking on glucose and lipid metabolism disturbances during pregnancy has been less studied [13,14,15]. Similarly, the available evidence of maternal alcohol consumption is particularly sparse [16,17]; among its main complications are cesarean delivery, stillbirth, high birth weight, and infant mortality.

Unhealthy lifestyles adopted by women of reproductive age also predispose them to overweight/obesity in pregnancy, associated with cardiometabolic risk factors such as insulin resistance [18] and worse lipid profile [19,20]. It has been suggested that inappropriate GWG, especially in later pregnancy, may also increase the risk of adverse obstetric outcomes [21,22].

Previous studies on maternal lifestyle behaviors and cardiometabolic risk during pregnancy have focused on specific cardiometabolic risk markers and only a few studies [5,23] have considered whether combinations of biological risk factors formed a clustered cardiometabolic risk (CCR) score. In this context, a cluster of cardiometabolic factors has been reported to be more strongly associated with adverse pregnancy outcomes than just one factor [24]. Using this factor-cluster approach would help to better identify high-risk women during pregnancy. It is also important to prospectively reassess the cardiometabolic risk of pregnant women in order to determine whether this risk is stable or whether it progresses over the course of pregnancy.

It can generally be stated that cardiometabolic risk markers during pregnancy are influenced by multiple factors specific to each population. However, few studies have been conducted specifically among pregnant populations in the Mediterranean area, where the socio-demographic and Mediterranean lifestyle traits of women can be regarded as protective factors against cardiovascular risks. The key to planning effective strategies to prevent and treat future obstetric complications is to understand which maternal factors have favorable effects on cardiometabolic risk during pregnancy and define critical periods in which this relationship is most affected.

To further knowledge in this area, we aimed to investigate the association between prenatal sociodemographic, lifestyle, and clinical characteristics and clustering cardiometabolic risk and its components in the first and third-trimester of pregnancy in a population of pregnant women from a Mediterranean region in northern Spain. 

## 2. Materials and Methods

### 2.1. Study Design

A population-based prospective cohort study of healthy pregnant women who participated in the ECLIPSES study was conducted from the first to the third-trimester of pregnancy. A description of ECLIPSES has been published elsewhere [25]. Eligible participants were healthy adult women over 18 years with ≤12 weeks of gestation. Details of the inclusion/exclusion criteria can be found elsewhere [25].

**Figure 1:**
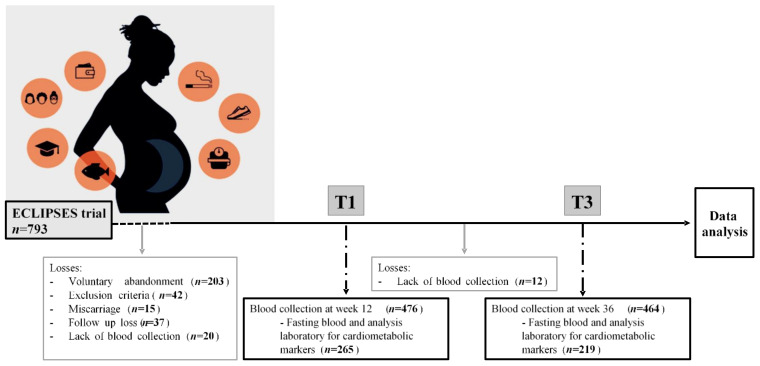
Flow chart of the study population.

Of the 793 pregnant women initially enrolled in the study, for the present analysis, all women who had data regarding serum cardiometabolic markers in the first (12 weeks) and/or third (36 weeks) trimester of pregnancy were included. The total study sample therefore comprised 265 pregnant women (Figure 1). All participants signed an informed consent form. The study was approved by the Ethical Committee of the Jordi Gol Institute for Primary Care Research and the Pere Virgili Institute for Health Research (approval ID: 118/2017. Date: 28 September 2017) and complied with the tenets of the Helsinki declaration.

### 2.2. Data Collection

Midwives and nutritionists collected the participants’ medical and obstetric history, gestational age, socioeconomic information, and education level. In the first and third-trimesters of pregnancy, lifestyle habits (PA, smoking, diet, and alcohol consumption), BP, and anthropometric measurements were also collected. The socioeconomic level was classified as low, mid, or high according to the Catalan classification of occupations (CCO-2011) [26]. Education level was classified as low (primary), medium (high school), and high (university studies or above). PA was measured using the short version of the International PA Questionnaire (IPAQ-S) [27]. Derived from total metabolic equivalents (METs-min/week) and based on the frequency and duration of walking and moderate and vigorous-intensity activity, this variable was divided into tertiles for analysis. The Fagerström questionnaire [28] was used to assess smoking, with women divided into three groups: current, former, and never smokers.

Eating habits were assessed through a self-administered food frequency questionnaire (FFQ) based on 45 food groups previously validated in our population [29]. Herein, we focused on women’s overall diet quality assessed using the relative rMedDiet score based on the intake of nine food groups [30]. This index, which was previously used in our published paper [30], is a modified version of the original MedDiet Score [31]. The resulting score ranged from 0 to 18 points, with larger values indicating greater diet quality. Since there are no pre-established cut-off points for the pregnant population, we divided the score into tertiles. Alcohol consumption was assessed as ‘yes’ or ‘no’.

Anthropometric measures were weight (kg) and height (cm). BMI was calculated from these measures (weight(kg)/height(m)^2^). Women were classified following WHO criteria [32] into normal weight (BMI 18.5–24.9 kg/m^2^), overweight (BMI 25.0–29.9 kg/m^2^), or obesity (BMI ≥ 30 kg/m^2^) in the first-trimester. Total GWG, calculated from the difference between the weights measured in the first and third-trimester visits and taking into account initial BMI, was categorized as insufficient, adequate, or excessive in accordance with 2009 IOM recommendations [33].

### 2.3. Cardiometabolic Risk Markers

Blood samples were collected at weeks 12 and 36 of pregnancy after an overnight fast and stored at −80 °C inside the Biobank until analysis. The fasting serum cardiometabolic biomarkers assessed included glucose, insulin, and lipids, which were analyzed at the accredited Laboratori Clínic ICS Camp de Tarragona-Terres de l’Ebre, Joan XXIII University Hospital in Tarragona (Spain). All samples were thawed and analyzed at the same time to minimize inter-batch variation. Simultaneously, glucose, total cholesterol, HDL cholesterol (HDL-c), and triglyceride (TG) concentrations were measured using standard enzymatic automated methods. Intra- and interassay coefficients of variation (CVs) were below 2.2% for all. LDL cholesterol (LDL-c) was calculated using the Friedewald formula (LDL-c = total cholesterol-HDL-c-triglycerides/5). Serum insulin levels were assayed by a chemiluminescent immunoassay method on an ADVIA Centaur analyzer using a commercial kit (ADVIA Centaur IRI, Siemens Healthcare Diagnostics Inc., Tarrytown, NY, USA). Lower and upper detection limits were 0.5 and 300 mUI/L, respectively. The intra- and interassay CV ranges were 3.3–4.6% and 2.6–5.9%, respectively. 

Insulin resistance was estimated by homeostasis model assessment (HOMA-IR) using the following equation: HOMA-IR = fasting insulin (μIU/mL) × fasting glucose (mmol/L)/22.5.

SBP and DBP were measured in both trimesters using an automatic digital monitor (Omron HEM-705CP).

A clustered cardiometabolic risk (CCR) score was created by summing all standardized z-scores (z = value-mean/SD of the whole population) of the seven cardiometabolic markers assessed (BMI, SBP, glucose, HOMA-IR index (log), TG (log), LDL-c, and HDL-c). HDL-c was calculated after values were multiplied by −1 since it is inversely related to metabolic risk. Only SBP was considered in the CCR score since SBP and DBP were highly correlated. A higher CCR score entails greater cardiometabolic risk. The rationale for selecting this CCR score and its components were based on a previous pregnancy study that used a similar risk score and factors [5].

The continuous CCR score was estimated for 264 women and 215 women whose seven health parameters were measured in the first and third trimester of pregnancy, respectively. In this study, the CCR score and each cardiometabolic factor were the primary and secondary outcomes, respectively.

### 2.4. Statistical Analysis

All statistical analyses were performed using the 15.0 version of STATA software (Stata Corp LP, College Station, TX, USA). Descriptive statistics were used to characterize the population. Data are expressed as mean ± SD for quantitative variables and number (%) for categorical variables. 

The normality of the data was tested using both statistical (Shapiro–Wilk test) and graphical methods (histograms and scatter plots). Variables non-normally distributed were logarithmically transformed for analyses (insulin, HOMA-IR, and TG). The between-group differences in each cardiometabolic risk variable in both trimesters were analyzed by one-way ANOVA with Bonferroni’s test for post hoc comparison and Student’s T-test, as appropriate. Paired-samples *t*-tests were performed to evaluate intra-group differences for the cardiometabolic risk variables between the first and the third trimesters.

Multivariable linear regression analyses were performed to evaluate the independent contributions of selected sociodemographic and lifestyle characteristics of the pregnant women on the CCR score and each cardiometabolic risk factor (BMI, SBP, DBP, glucose, insulin, HOMA-IR, TG, HDL-c, LDL-c) in the first and third trimesters of pregnancy. A multivariable linear regression analysis was also performed to evaluate the independent contribution of the first-trimester CCR score to the third-trimester CCR score. We used our prior knowledge to select the following prenatal characteristics: age (<25, 25–29, ≥30 years), social class (lower/medium, high), education level (primary/secondary, university studies), smoking status (non-smoker, current/former smoker), alcohol consumption (no, yes) PA (METs-min/week, tertiles), rMedDiet score (tertiles), and GWG (insufficient, adequate, excessive). Estimates were presented as β coefficient (β) and 95% confidence intervals (CIs). Multicollinearity was assessed by inspecting the tolerance (1/VIF) values and variance inflation factors (VIFs) for this multivariable model. All tolerance values were above 0.7 and all VIFs were below 2.0, which suggests there were no concerns over multicollinearity. Statistical significance was set at *p* < 0.05.

## 3. Results

The sociodemographic and lifestyle characteristics of pregnant women are shown in Table 1. The mean age of the women was 29.6 (SD, 4.7), with 57% of them over 30 years old. Their mean initial BMI was 24.1 (3.5) kg/m2, with roughly 36% of them classified as overweight/obese with a BMI ≥25.0 kg/m2. Their mean GWG was 10.4 (3.6) kg. According to IOM recommendations, 37% of the women met the criteria for GWG, while 45% fell below them and 18% exceeded them. A third of the women (32%) had received a university education, 19% of them were from a high social class, and 31% were former smokers or smoked during pregnancy. Mean PA was 475.8 (701.9) METs-min/week and the mean rMedDiet score was 9.4 (2.4).

All cardiometabolic markers and lipid parameters increased between the first and third trimesters, while fasting glucose decreased (all *p* < 0.05).

Comparisons between the characteristics of pregnant women in relation to their CCR score and its components between the first and third-trimesters are shown in Supplementary Tables S1 and S2, respectively. Results from multivariate-adjusted regression analyses in the first trimester are shown in Table 2. These cross-sectional analyses showed that, irrespective of other factors: age above 30 years was significantly associated with greater HDL-c levels; university education was associated with lower BMI and SBP; and a higher level of PA was associated with lower LDL-c levels (all *p* < 0.05). Multiple regression analysis, on the other hand, showed that obese/overweight status in early pregnancy was, as expected, independently and positively associated with BMI, SBP, DBP, insulin, HOMA-IR, and LDL-c levels (all *p* < 0.05).

Prospective multivariate-adjusted analyses (Table 3) showed that the associations between overweight/obesity status and higher BMI and lower HDL-c levels persisted in the third-trimester even after potential confounders were controlled (all *p* < 0.05). Similarly, BMI and SBP levels were higher in women with excessive GWG, while HDL-c levels increased. Moreover, multivariate analysis showed that smoking and drinking alcohol in pregnancy were independent factors associated with fasting TG and LDL-c, and both SBP and DBP, respectively, as time progressed. However, BMI, SBP and DBP levels and fasting glucose concentrations showed a significant inverse association with insufficient GWG. Additionally, women with a university education showed smaller increases in BMI during their pregnancy, while high social class was inversely associated with lower fasting glucose, insulin, and HOMA-IR levels at the end of pregnancy (all *p* < 0.05).

**Figure 2:**
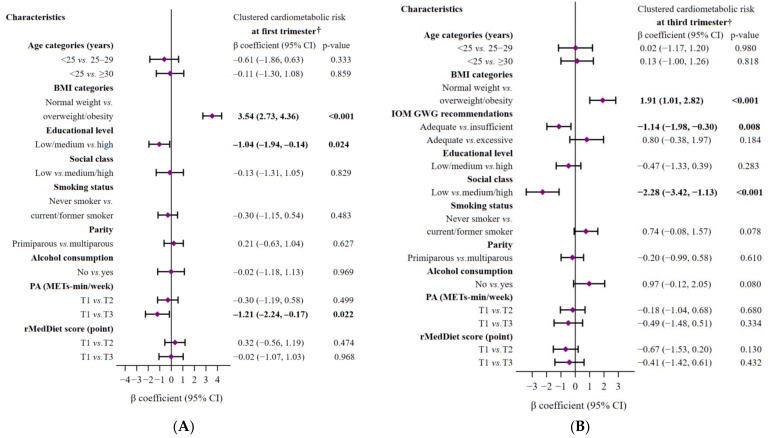
Multivariate-adjusted linear regression models of the associations between sociodemographic and lifestyle characteristics of pregnant women and clustered cardiometabolic risk in the first (**A**) and third (**B**) trimesters of pregnancy. The models were mutually adjusted for all characteristics displayed in each figure. The diamonds represent the β coefficient (β), and the whisker plots represent 95% CIs. The significance of the numbers in bold is *p*-value < 0.05. Abbreviations: BMI, body mass index; GWG, gestational weight gain; IOM, Institute of Medicine; PA, Physical Activity; METs, metabolic equivalents; T, tertile; rMedDiet, Mediterranean diet. ^†^ A higher clustered cardiometabolic status signifies higher cardiometabolic risk.

Figure 2, which shows subgroup analyses by different variables of interest, reveals statistically significant associations between CCR scores and overweight/obesity status (positive), university education (negative), and higher levels of PA (negative) at the beginning of pregnancy (all *p* < 0.05). Note that the associations between overweight/obesity status and CCR score persisted into the third-trimester. Moreover, a significant association was found between women with insufficient GWG and those with high social class and lower CCR scores (all *p* < 0.05). In the third-trimester, no significant association with other factors was found.

After adjusting for confounding factors, we found that the first-trimester CCR score was significantly and independently related to the third trimester CCR score (β: 0.31, 95%CI: 0.19, 0.43; *p* < 0.001).

## 4. Discussion

This study describes the association between maternal factors (socio-demographic and lifestyle characteristics) and clustering cardiometabolic risk and its components throughout pregnancy in a Spanish population of healthy pregnant women. Our main findings are that potentially modifiable prenatal factors, such as having a normal weight in early pregnancy, lower GWG, and more PA, as well as higher education and social class levels, were significantly and independently associated with lower CCR. Smoking and drinking alcohol during pregnancy also showed a non-significant trend towards higher CCR at the end of pregnancy. The results of each cardiometabolic biomarker also maintained the same relationship. Interestingly, the women’s CCR score in the first trimester was an independent predictor of their CCR score in the third trimester, which suggests cardiometabolic risk progressed as pregnancy advanced.

We can hypothesize from our findings that BMI at pregnancy baseline is more relevant than GWG when predicting cardiometabolic risk during pregnancy. Indeed, we found that early pregnancy overweight/obesity was the strongest predictor of the CCR score in both early and late pregnancy. Despite the importance of maternal obesity for the subsequent development of cardiovascular and metabolic alterations, to our knowledge, this is the first time that this relationship has been described in pregnant women using a composite risk score. Moreover, overweight/obese women had a less favorable cardiometabolic profile, with higher SPB, DPB, insulin resistance, and LDL-c in the first-trimester than their normal-weight counterparts. As our results and those of other studies conducted in the first-trimester of pregnancy show, being overweight/obese increases the risk of hypertension in pregnant women [34,35].

We found that SBP and DBP in women with insufficient GWG decreased in the third-trimester. These findings are consistent with a recent meta-analysis of observational studies, which showed that excessive GWG is associated with a higher risk of hypertensive disorders during pregnancy [36] and should therefore be avoided.

Our data support previous evidence that showed that overweight/obese pregnant women had significantly higher insulin and HOMA-IR, especially in the first-trimester [37]. However, the effect of GWG on glucose metabolism is less studied and the few data published are somewhat contradictory [38,39,40]. In the present study, women who did not gain enough weight during pregnancy had lower blood glucose levels in the third trimester than those with adequate weight gain. It has been argued that, just like outside pregnancy, an increase in maternal adiposity during pregnancy causes a higher systemic inflammatory response and greater oxidative stress, which in turn promote hyperglycemia and, eventually, insulin resistance [41,42].

Serum lipid concentrations are known to increase as pregnancy progresses [19,43,44]. However, this pregnancy-associated hyperlipidemia appears to be exacerbated in overweight/obese women, probably as a result of insulin resistance [39,45,46,47]. In line with previous studies [45,46], our data suggest that overweight/obese pregnant women are more likely to present a more pro-atherogenic lipid profile. Our data also showed a positive association between excessive GWG and a significant increase in HDL-c in the third-trimester. In accordance with this observation, a recent study suggested that high levels of HDL-c in the third-trimester, especially in women with excessive GWG, may be considered a high-risk indicator of small size for gestational age [48].

From our findings and the above evidence, it is imperative that overweight/obese women of reproductive age should be encouraged to undertake preconception-intensive behavioral lifestyle interventions for weight loss and improve their metabolic status before and during very early pregnancy [49]. As Catalano suggests [50], unfavorable maternal status in terms of weight or cardiometabolic profile in early pregnancy is a harbinger of future abnormalities in late pregnancy and beyond. Those findings are also supported by our study, which found a significant association between first and third-trimester CCR scores.

With regard to lifestyle factors such as diet, there is clear evidence that certain individual nutrients and food groups are associated with cardiovascular risk also in the pregnant population [5,6,7,8]. However, our study did not show a relationship between the quality of the maternal diet (using the Mediterranean diet score) and cardiometabolic risk during pregnancy. Nevertheless, our results support the importance of adhering to this healthy dietary pattern since it protects against maternal obesity, excessive GWG, and other adverse short-term and long-term maternal and child outcomes [51]. A more specific study focused on individual dietary components (nutrients or food groups) could establish a relationship.

Similar to other Spanish studies [52], 13% of the pregnant women in our study consumed alcohol. Our findings support previous results [16] that showed that in the third-trimester, SBP, DBP, and LDL-c were higher in women who consumed alcohol than in those who did not.

Exposure to tobacco smoke during pregnancy also influences lipid-profile parameters. We also found that pregnant smokers had significantly higher third trimester levels of TG and LDL-c, even after adjusting for BMI and GWG, as well as a tendency towards a worse cardiometabolic risk profile. The two epidemiological studies conducted in this field so far have also revealed a more unfavorable lipid profile in pregnant smokers than in pregnant non-smokers [14,15]. Increased lipoprotein lipase (LPL) activity may be responsible for elevated LDL-c levels through the LPL-mediated degradation of TG-rich chylomicrons and VLDL, which, probably induced by nicotine, is markedly higher in smokers [53]. Another effect of nicotine on lipid metabolism is impaired LDL-c clearance [54]. Moreover, nicotine also increases circulating free fatty acid through enhanced lipolysis resulting from sympathoadrenal stimulation [55]. Thus, smoking and the presence of lipid disorders are inadvisable during pregnancy since they may also contribute to deleterious cardiovascular and atherogenic effects.

With regard to maternal PA, our results agree with those of earlier studies which suggest that habitual PA reduces TG and total cholesterol during early pregnancy [56,57], and LDL-c in the last two trimesters [56,57]. This highlights the importance of promoting PA to control lipid disorders, especially in the first-trimester when the fetal organs are formed, and the placenta begins to develop [58].

In the present study, socio-environmental factors, especially higher levels of education (in relation to lower BMI and SBP) and social class (in relation to lower fasting glucose, insulin, and HOMA-IR) were also strongly associated with better cardiometabolic markers and lower CCRs in the first and third-trimesters of pregnancy, respectively. Generally, these findings are supported by those of previous studies [3,59,60]. However, the nature of such associations during pregnancy remains unclear. They probably reflect a combination of social/psychological factors and healthier behaviors (in those with higher education) that result directly or indirectly in cardiometabolic benefits. We found, for example, that pregnant women with higher social and educational levels were older and had lower early pregnancy BMI. In the present study, these factors appear to be associated with a better metabolic phenotype during gestation.

The main strength of our study is the analysis of cardiometabolic health during pregnancy using a clustering of cardiometabolic risk factors, which provides greater overall risk than any individual factor on its own. This approach has rarely been used in previous studies. Moreover, we decided to use a CCR score that combined clinical and biochemical parameters, including adiposity, BP, insulin resistance, and lipids, since these can be measured easily in routine clinical practice and, even more importantly, are all major risk factors for cardiovascular disease. Additionally, the continuous CCR score is statistically more sensitive and less prone to error than categorical forms. Another advantage of our study is its prospective design and relatively large sample size, reinforcing the usefulness of our results. However, certain study limitations should also be considered. Namely, we use the maternal final weight at around 36 weeks to calculate GWG, which may cause misclassification of the GWG category, especially among overweight/obese women, thus reducing the estimated effect. Additionally, the CMR score is specific to this study sample, and we assumed that each component has equal weight in predicting metabolic risk. 

## 5. Conclusions

Our findings provide evidence of the effects of sociodemographic, lifestyle, and clinical characteristics during pregnancy on cardiometabolic health in a Spanish Mediterranean population of healthy pregnant women. The most protective modifiable prenatal factors of cardiometabolic risk during pregnancy were being of normal weight, having higher levels of education and social class, engaging in greater PA, being a non-smoker, and not drinking alcohol. These findings will help policymakers to improve metabolic status in women before and/or during very early pregnancy in order to prevent obstetric complications. Further prospective studies are needed to determine whether clustering cardiometabolic risk variables helps to determine the risk of adverse mother and fetus/child outcomes more than individual risk factors.

## Figures and Tables

**Table 1:** Sociodemographic and lifestyle characteristics of pregnant women in the first trimester of pregnancy (n = 265).

General Characteristics	Summary Statistics
Age (years), mean ± SD	29.6 ± 4.7
Age categories (years), n (%)	
<25	40 (15)
25–29	73 (28)
≥30	152 (57)
Weight (kg), mean ± SD	63.3 ± 9.6
BMI (kg/m^2^), mean ± SD	24.1 ± 3.5
BMI categories, n (%)	
18.5–24.9 (normal weight)	169 (64)
25.0–29.9 (overweight)	82 (31)
≥30 (obesity)	14 (5)
GWG (kg), mean ± SD	10.4 ± 3.6
IOM GWG recommendations, n (%) ^†^	
Insufficient	119 (45)
Adequate	99 (37)
Excessive	47 (18)
Educational level, n (%)	
Low (primary or below)	83 (31)
Medium (secondary)	97 (37)
High (university or above)	84 (32)
Social class, n (%)	
Low	35 (13)
Medium	180 (68)
High	49 (19)
Smoking status, n (%)	
Never smoker	185 (70)
Former smoker	42 (16)
Current smoker	37 (14)
Alcohol consumption	
No	222 (87)
Yes	33 (13)
Physical Activity (METs-min/week)	
T1 (<1070)	87 (33)
T2 (1070–3336)	117 (44)
T3 (≥3336)	60 (23)
rMedDiet score (point)	
T1 (<9)	92 (36)
T2 (9–12)	107 (42)
T3 (≥12)	56 (22)

Values are expressed in means ± SD (standard deviation) or number (%, percentage). Abbreviations: BMI, body mass index; GWG, gestational weight gain; IOM, Institute of Medicine; METs, metabolic equivalents; T, tertile; rMedDiet, Mediterranean diet; ^†^ Recommendations for GWG according to IOM guidelines are: initial BMI < 18.5 kg/m^2^, total weight gain 12.5–18 kg; BMI 18.5–24.9 kg/m^2^, total weight gain 11.5–16 kg; BMI 25.0–29.9 kg/m^2^, total weight gain 7–11.5 kg; and BMI ≥ 30 kg/m^2^, total weight gain 5–9 kg.

**Table 2:** Multivariate-adjusted linear regression models of the associations between sociodemographic and lifestyle characteristics ^†^ of pregnant women and cardiometabolic risk markers in the first trimester of pregnancy.

	Cardiometabolic Risk Markers in the First Trimester																	
	BMI (kg/m^2^)		SBP (mm Hg)		DBP (mm Hg)		Glucose (mg/dL)		Insulin (mU/L) ^‡^		HOMA-IR ^‡^		Triglycerides (mg/dL) ^‡^		HDL-c (mg/dL)		LDL-c (mg/dL)	
Characteristics	β	*p*	β	*p*	β	*p*	β	*p*	β	*p*	β	*p*	β	*p*	β	*p*	β	*p*
Age categories (years)																		
<25 vs. 25–29	−0.09	0.843	1.62	0.491	1.73	0.269	2.97	0.188	−0.20	0.085	−0.16	0.208	−0.13	0.095	4.88	0.068	−1.90	0.714
<25 vs. ≥30	0.04	0.920	2.02	0.371	1.93	0.199	3.34	0.123	−0.08	0.442	−0.04	0.731	−0.05	0.508	6.93	0.007 **	2.24	0.652
BMI categories																		
Normal weight vs.	5.83	<0.001 **	5.42	0.001 **	3.64	<0.001 **	1.95	0.188	0.30	<0.001 **	0.33	<0.001 **	0.09	0.067	−2.56	0.144	6.80	0.045 *
overweight/obesity																		
Educational level																		
Low/medium vs. high	−0.68	0.030 *	−4.12	0.017 *	−1.61	0.158	−1.46	0.373	0.01	0.923	−0.01	0.937	−0.08	0.162	2.92	0.132	0.31	0.934
Social class																		
Low vs. medium/high	−0.05	0.889	1.96	0.383	0.06	0.969	0.23	0.916	−0.18	0.107	−0.16	0.186	−0.00	0.998	0.17	0.947	1.18	0.811
Smoking status																		
Never smoker vs.	−0.20	0.489	−2.62	0.103	−0.99	0.353	2.33	0.130	−0.05	0.492	−0.01	0.893	−0.09	0.081	−1.54	0.395	−3.63	0.303
current/former smoker																		
Alcohol consumption																		
No vs. yes	0.02	0.970	3.21	0.144	1.70	0.243	−0.44	0.834	−0.08	0.473	−0.07	0.529	−0.04	0.547	0.48	0.845	0.97	0.840
PA (METs-min/week)																		
T1 vs. T2	0.05	0.875	−0.32	0.851	−1.50	0.181	0.46	0.775	0.017	0.839	0.01	0.871	−0.06	0.307	0.11	0.954	−2.98	0.420
T1 vs. T3	−0.57	0.113	−0.94	0.632	−1.45	0.265	0.85	0.648	−0.08	0.421	−0.07	0.497	−0.12	0.068	2.38	0.282	−9.83	0.023 *
rMedDiet score (point)																		
T1 vs. T2	−0.31	0.308	−1.33	0.422	−0.32	0.770	0.74	0.643	−0.01	0.968	0.00	0.993	0.09	0.087	−0.63	0.738	3.02	0.407
T1 vs. T3	−0.45	0.216	−0.20	0.919	−0.30	0.823	1.32	0.487	−0.09	0.357	−0.07	0.517	0.11	0.077	0.48	0.829	−3.78	0.387

Multivariate linear regression models were used to calculate the β coefficient (β). The models were run separately for each cardiometabolic risk marker. The models were mutually adjusted for all characteristics displayed in this table. Abbreviations: BMI, body mass index; PA, Physical Activity; METs, metabolic equivalents; T, tertile; rMedDiet, Mediterranean diet; SBP, systolic blood pressure; DBP, diastolic blood pressure; HOMA-IR, Homeostatic Model Assessment for Insulin Resistance; HDL-c, high-density lipoprotein-cholesterol; LDL-c, low-density lipoprotein-cholesterol. ^†^ In the first trimester of pregnancy. ^‡^ Natural log-transformed values. * The significance of the numbers in bold is *p*-value < 0.05. ** The significance of the numbers in bold is *p*-value < 0.001.

**Table 3:** Multivariate-adjusted linear regression models of the associations between sociodemographic and lifestyle characteristics ^†^ of pregnant women and cardiometabolic risk markers in the third trimester of pregnancy.

	Cardiometabolic Risk Markers in the Third Trimester																	
	BMI (kg/m^2^)		SBP (mm Hg)		DBP (mm Hg)		Glucose (mg/dL)		Insulin (mU/L) ^‡^		HOMA-IR ^‡^		Triglycerides (mg/dL) ^‡^		HDL-c (mg/dL)		LDL-c (mg/dL)	
Characteristics	β	*p*	β	*p*	β	*p*	β	*p*	β	*p*	β	*p*	β	*p*	β	*p*	β	*p*
Age categories (years)																		
<25 vs. 25–29	0.50	0.225	−0.92	0.705	−0.41	0.822	2.48	0.276	−0.15	0.264	−0.11	0.444	−0.02	0.844	3.49	0.278	6.05	0.456
<25 vs. ≥30	0.50	0.200	0.25	0.913	−0.69	0.696	3.03	0.164	−0.19	0.140	−0.15	0.283	−0.04	0.712	3.07	0.317	8.81	0.256
BMI categories																		
Normal weight vs. over	4.14	<0.001 **	−0.79	0.669	2.33	0.097	−1.99	0.252	0.16	0.120	0.11	0.305	0.11	0.180	−5.10	0.039 *	−1.21	0.845
weight/obesity																		
IOM GWG recommendations																		
Adequate vs. insufficient	−1.32	<0.001 **	-4.07	0.018 *	-3.45	0.008 *	-3.38	0.036 *	-0.05	0.584	-0.10	0.340	0.09	0.229	0.90	0.690	−0.27	0.962
Adequate vs. excessive	2.15	<0.001 **	5.76	0.017 *	1.01	0.580	-0.58	0.795	0.10	0.448	0.11	0.441	0.04	0.730	8.02	0.012 *	4.46	0.576
Educational level																		
Low/medium vs. high	−0.58	0.053	0.16	0.929	−0.74	0.577	−1.28	0.436	−0.09	0.323	−0.11	0.297	−0.08	0.330	2.23	0.336	1.12	0.060
Social class																		
Low vs. medium/high	−0.38	0.342	−1.78	0.447	−0.64	0.719	−5.87	0.008 *	−0.40	0.002 *	−0.49	<0.001 **	−0.15	0.153	2.32	0.454	−7.57	0.335
Smoking status																		
Never smoker vs.	0.29	0.311	−0.43	0.799	1.27	0.321	−0.77	0.625	0.09	0.304	0.08	0.395	0.18	0.016 *	3.07	0.168	1.19	0.034 *
current/former smoker																		
Alcohol consumption																		
No vs. yes	−0.20	0.586	4.74	0.032 *	3.57	0.034 *	1.41	0.493	−0.02	0.859	0.01	0.959	−0.00	1.000	−1.94	0.503	1.57	0.032 *
PA (METs-min/week)																		
T1 vs. T2	0.19	0.523	1.99	0.257	−1.15	0.388	1.33	0.414	0.00	0.960	0.03	0.789	−0.09	0.230	1.54	0.504	−2.35	0.685
T1 vs. T3	−0.52	0.130	0.62	0.760	−1.72	0.263	−1.75	0.356	0.13	0.237	0.09	0.465	−0.01	0.877	0.67	0.801	−4.47	0.508
rMedDiet score (point)																		
T1 vs. T2	0.21	0.491	−1.27	0.470	0.66	0.624	−1.99	0.226	−0.02	0.819	−0.06	0.536	0.05	0.562	3.66	0.117	−7.02	0.233
T1 vs. T3	−0.19	0.587	1.42	0.494	0.14	0.931	−0.84	0.665	−0.11	0.339	−0.12	0.333	0.06	0.527	2.12	0.438	−2.89	0.675

Multivariate linear regression models were used to calculate the β coefficient (β). The models were mutually adjusted for all characteristics displayed in this table. Abbreviations: BMI, body mass index; GWG, gestational weight gain; IOM, Institute of Medicine; PA, Physical Activity; METs, metabolic equivalents; T, tertile; rMedDiet, Mediterranean diet; SBP, systolic blood pressure; DBP, diastolic blood pressure; HOMA-IR, Homeostatic Model Assessment for Insulin Resistance; HDL-c, high-density lipoprotein-cholesterol; LDL-c, low-density lipoprotein-cholesterol. ^†^ In the first trimester of pregnancy (except for GWG). ^‡^ Geometric means of log-transformed values. * The significance of the numbers in bold is *p*-value < 0.05. ** The significance of the numbers in bold is *p*-value < 0.001.

## Data Availability

The datasets generated and/or analyzed during the current study are not publicly available due to subject confidentiality but are available from the corresponding author on reasonable request.

## Supplementary Materials

The following supporting information can be downloaded at: https://www.mdpi.com/article/10.3390/nu15051135/s1, Table S1: Comparisons of selected sociodemographic and lifestyle characteristics† of pregnant women for single risk factors and cardiometabolic risk scores in the first trimester of pregnancy. Table S2: Comparisons of selected sociodemographic and lifestyle characteristics† of pregnant women for single risk factors and cardiometabolic risk scores in the third trimester of pregnancy.
