# Supplementary material for: Prenatal Factors Associated with Maternal Cardiometabolic Risk Markers during Pregnancy: The ECLIPSES Study
Source: Nutrients. 2023 Feb 23;15(5):1135. doi: 10.3390/nu15051135 (PMC10005748; doi:10.3390/nu15051135)
Supplement: Supplementary file 1 [file nutrients-15-01135-s001.zip › nutrients-2198832-supplementary.pdf]

Supplementary Table S1. Comparisons of selected sociodemographic and lifestyle characteristics† of pregnant women for single risk factors and cardiometabolic risk scores in the first trimester of pregnancy

	**Cardiometabolic risk biomarkers in the first trimester**									
	BMI (kg/m^2)	SBP (mm Hg)	DBP (mm Hg)	Glucose (mg/dL)	Insulin (mU/L) ‡	HOMA-IR‡	Triglycerides (mg/dL) ‡	HDL-c (mg/dL)	LDL-c (mg/dL)	CCR-z score§
	n=265	n=264[a]	n=264	n=265	n=265	n=265	n=265	n=265	n=265	n=264
**Characteristics**	Mean ± SD	Mean ± SD	Mean ±SD	Mean ± SD	Mean ± SD	Mean ± SD	Mean ± SD	Mean ± SD	Mean ± SD	Mean ± SD
All	24.1 ± 3.5	112.3 ± 11.8	66.4 ± 7.7	70.2 ± 10.7	7.77 ± 1.76	1.32 ± 1.85	83.1 ± 1.4	61.2 ± 13.0	88.2 ± 25.5	-1.5x10^{-3} ± 3.54
Age categories (years)										
<25	23.9 ± 4.4	111.5 ± 13.0	64.9 ± 7.7	67.7 ± 10.6	8.25 ± 1.80	1.36 ± 1.93	85.6 ± 1.5	55.9 ± 11.5	86.8 ± 28.9	0.07 ± 3.66
25-29	23.7 ± 3.0	111.7 ± 12.8	66.1 ± 7.6	70.4 ± 11.3	6.89 ± 1.71	1.18 ± 1.84	79.0 ± 1.5	61.4 ± 14.0	85.1 ± 22.3	-0.59 ± 3.42
≥30	24.3 ± 3.4	112.8 ± 11.0	66.9 ± 7.7	70.8 ± 10.4	8.00 ± 1.78	1.39 ± 1.85	83.9 ± 1.4	**62.6 ± 12.5***	90.0 ± 25.9	0.26 ± 3.55
BMI categories (kg/m^2)										
<25 (normal weight)	21.9 ± 1.7	110.3 ± 11.7	65.0 ± 7.5	69.5 ± 10.4	6.89 ± 1.75	1.16 ± 1.84	79.0 ± 1.4	62.1 ± 13.0	85.6 ± 24.1	-1.33 ± 2.94
≥25 (overweight /obesity)	**27.8 ± 1.7****	**115.9 ± 11.7****	**68.9 ± 7.5****	71.4± 10.4	**9.49 ± 1.69****	**1.64 ± 1.78****	**89.1 ± 1.4***	59.7 ± 13.0	**92.8 ± 25.2***	**2.36 ± 3.27***
Educational level										
Low (primary or below)/ Medium (secondary)	24.5 ± 3.5	113.6 ± 11.9	66.8 ± 7.6	70.5 ± 11.4	8.00 ± 1.78	1.37 ± 1.89	84.7 ± 1.4	59.7± 13.2	87.6 ± 20.0	0.45 ± 3.41
High (university or above)	**23.2 ± 3.3***	**109.6 ± 11.1***	65.5 ± 7.9	69.5 ± 9.0	7.24 ± 1.73	1.23 ± 1.78	75.9 ± 1.5	**64.6 ± 12.1***	89.3 ± 34.5	**-0.99 ± 3.64***
Social class										
Low	25.4 ± 3.8	112.6 ± 13.9	67.2 ± 6.4	70.5 ± 15.6	9.97 ± 1.71	1.68 ± 1.85	87.3 ± 1.5	58.3 ± 12.9	86.7 ± 18.8	1.18 ± 3.13
Medium/high	**23.9 ± 3.4***	112.3 ± 11.5	66.3 ± 7.9	70.1 ± 9.8	**7.46 ± 1.76***	**1.27 ±1.85***	81.4 ± 1.4	61.7 ± 13.0	88.4 ± 26.4	**-0.18 ± 3.57***
Smoking status										
Never smoker	24.2 ± 3.7	112.9 ± 12.0	66.7 ± 7.5	69.6 ± 11.4	8.00 ± 1.80	1.34 ± 1.91	84.7 ± 1.5	61.6 ± 13.7	89.6 ± 27.3	0.18 ± 3.81
Current/former smoker	23.7 ± 3.0	110.9 ± 11.4	65.5 ± 8.2	71.5 ± 8.5	7.32 ± 1.71	1.28 ± 1.76	**75.9 ± 1.4***	60.4 ± 11.3	84.8 ± 20.4	-0.44 ± 2.78
Alcohol consumption										
No	24.2 ± 3.6	112.0 ± 11.4	66.4 ± 7.4	70.3 ± 11.1	7.92 ± 1.80	1.34 ±1.89	83.0 ± 1.5	61.1 ± 13.0	88.1 ± 24.7	0.08 ± 3.68
Yes	23.6 ± 3.0	114.2 ± 13.8	67.5 ± 9.5	69.9 ± 8.5	7.32 ± 1.68	1.25 ± 1.71	78.2 ± 1.4	61.9 ± 13.8	89.3 ± 30.1	-0.26 ± 2.96
PA (METs-min/week)										
T1	24.3 ± 3.6	112.8 ± 11.4	67.6 ± 7.7	69.9 ± 8.9	7.92 ± 1.78	1.36 ± 1.89	87.3 ± 1.4	61.1 ± 13.0	91.8 ± 28.6	0.47 ± 3.60
T2	24.4 ± 3.6	112.7 ± 12.6	65.9 ± 7.9	70.2 ± 11.6	8.00 ± 1.80	1.37 ± 1.87	83.0 ± 1.4	60.5 ± 13.3	89.1 ± 23.6	0.23 ± 3.61
T3	**23.1 ± 3.0***	110.9 ± 10.9	65.6 ± 7.2	70.6 ± 11.4	7.03 ± 1.69	1.20 ± 1.78	75.9 ± 1.4	62.9 ± 12.4	**80.9 ± 22.9***	**-1.14 ± 3.11***
rMedDiet score (point)										
T1	24.4 ± 4.0	113.0 ± 11.7	66.7 ± 7.3	69.7 ± 10.0	8.08 ± 1.82	1.37 ± 1.85	78.25 ± 1.4	60.8 ± 11.8	87.8 ± 20.9	0.03 ± 3.45
T2	23.7 ± 3.1	111.4 ± 12.1	66.2 ± 7.6	70.2 ± 11.9	7.85 ± 1.73	1.33 ± 1.82	83.93 ± 1.4	61.2 ± 13.4	90.3 ± 31.5	-0.01 ± 3.80

T3	24.3 ± 3.4	112.8 ± 11.1	66.8 ± 8.7	71.2 ± 10.0	7.39 ± 1.84	1.29 ± 2.01	87.35 ± 1.4	61.9 ± 14.5	85.1 ± 18.2	0.14 ± 3.44

Values are expressed in means ± SD (standard deviation). Abbreviations: BMI, body mass index; PA, Physical Activity; METs, metabolic equivalents; T, tertile; rMedDiet, Mediterranean diet; SBP, systolic blood pressure; DBP, diastolic blood pressure; HOMA-IR, Homeostatic Model Assessment for Insulin Resistance; HDL-c, high-density lipoprotein-cholesterol; LDL-c, low-density lipoprotein-cholesterol; CCR, clustered cardiometabolic risk. The significance of the numbers in bold is p-value < 0.05. * p<0.05 and ** p<0.001 compared with the first category as derived from ANOVA or Student's T-test, as appropriate. †In the first trimester of pregnancy. ‡Geometric means of log-transformed values. §A higher clustered cardiometabolic status signifies higher cardiometabolic risk.

Supplementary Table S2. Comparisons of selected sociodemographic and lifestyle characteristics† of pregnant women for single risk factors and cardiometabolic risk scores in the third trimester of pregnancy

	**Cardiometabolic risk biomarkers in the third trimester**									
**Characteristics**	BMI (kg/m^2) n=219 Mean ± SD	SBP (mm Hg) n=215 Mean ± SD	DBP (mm Hg) n=215 Mean ±SD	Glucose (mg/dL) n=219 Mean ± SD	Insulin (mU/L) ‡ n=219 Mean ± SD	HOMA-IR‡ n=219 Mean ± SD	Triglycerides (mg/dL) ‡ n=219 Mean ± SD	HDL-c (mg/dL) n=219 Mean ± SD	LDL-c (mg/dL) n=219 Mean ± SD	CCR-z score§ n=215 Mean ± SD
All	27.8 ± 3.3	113.7 ± 11.0	68.8 ± 8.6	67.3 ± 10.0	8.58 ± 1.82	1.40 ± 1.91	170.7 ± 1.6	65.7 ± 14.1	131.2 ± 35.8	2.2x10^{-3} ± 3.12
Age (years)										
<25	27.1 ± 3.1	114.58 ± 12.3	69.9 ± 8.8	66.7 ± 7.5	10.38 ± 1.71	1.70 ± 1.80	172.4 ± 1.6	63.0 ± 12.4	123.1 ± 42.6	0.14 ± 3.12
25-29	27.8 ± 3.3	112.97 ± 10.9	69.2 ± 8.2	67.3 ± 9.6	8.67 ± 1.91	1.43 ± 2.01	170.7 ± 1.6	67.0 ± 14.6	128.4 ± 30.9	-0.15 ± 3.03
≥30	27.9 ± 3.3	113.90 ± 10.8	68.4 ± 8.8	67.5 ± 10.8	8.08 ± 1.78	1.34 ± 1.88	170.7 ± 1.7	65.7 ± 14.3	134.7 ± 36.0	0.04 ± 3.18
BMI (kg/m^2)										
<25 (normal weight)	25.9 ± 2.0	112.7 ± 10.7	67.7 ± 8.1	67.2 ± 9.06	8.00 ± 1.85	1.31 ± 1.93	164.0 ± 1.6	66.7 ± 14.2	131.3 ± 34.2	-0.96 ± 2.78
≥25 (overweight/obesity)	**31.9 ± 2.3**[**]	115.5 ± 11.4	**70.9 ± 9.2**[*]	67.5 ± 11.6	**9.77 ± 1.69**[*]	**1.60 ± 1.84**[*]	183.1± 1.6	63.75 ± 13.7	131.0 ± 38.8	**1.81 ± 2.91**[*]
IOM GWG recommendations¶										
Insufficient	25.9 ± 2.3	110.6 ± 9.6	66.3 ± 8.3	65.9 ± 9.4	7.84 ± 1.84	1.27 ± 1.93	170.7 ± 1.6	66.3 ± 13.1	130.2 ± 34.2	-1.21 ± 2.77
Adequate	**28.2 ± 2.6**[**]	**114.6 ± 11.2**[*]	**70.4 ± 8.2**[*]	69.1 ± 11.4	8.75 ± 1.87	1.46 ± 1.99	165.7 ± 1.7	63.2 ± 14.3	132.2 ± 38.4	**0.64 ± 3.19**[*]
Excessive	**32.1 ± 2.7**[**]	**120.6 ± 11.3**[**]	**72.9 ± 8.2**[**]	67.4 ± 7.7	**10.60 ± 1.55**[*]	**1.75 ± 1.58**[a]	181.3 ± 1.5	69.4 ± 15.6	132.0 ± 35.5	**2.12 ± 2.27**[*]
Educational level										
Low (primary or below)/ Medium (secondary)	28.2 ± 3.2	113.9 ± 11.1	69.0 ± 8.6	68.0 ± 10.1	9.20 ± 1.80	1.54 ± 1.92	177.7 ± 1.6	64.8 ± 14.7	128.0 ± 33.3	0.38 ± 2.94
High (university or above)	**27.1 ± 3.4**[*]	113.3 ± 11.0	68.5 ± 8.7	66.0 ± 9.9	**7.31 ± 1.80**[*]	**1.19 ± 1.86**[*]	**154.5 ± 1.7**[*]	67.4 ± 12.8	**138.2 ± 40.0**[*]	**-0.76 ± 3.32**[*]
Social class										
Low	29.0 ± 2.8	115.0 ± 12.7	69.6 ± 8.3	72.5 ± 11.2	13.06 ± 1.69	2.32 ± 1.84	200.3 ± 1.3	61.5 ± 9.8	130.8 ± 31.4	2.38 ± 2.87
Medium/high	**27.6 ± 3.3**[*]	113.5 ± 10.8	68.7 ± 8.7	**66.6 ± 976**[*]	**8.08 ± 1.78**[*]	**1.31 ± 1.86**[*]	**165.7 ± 1.6**[*]	66.3 ± 14.6	131.4 ± 36.6	**-0.35 ± 3.00**[*]
Smoking status										
Never smoker	27.6 ± 3.2	113.3 ± 11.3	68.5 ± 7.8	67.4 ± 10.5	8.24 ± 1.89	1.36 ± 1.99	162.4 ± 1.7	64.8 ± 13.6	127.9 ± 33.5	-0.26 ± 3.10
Current/former smoker	28.1 ± 3.6	114.7 ± 10.3	69.7 ± 10.4	67.2 ± 8.8	9.29 ± 1.61	1.54 ± 1.68	**192.5 ± 1.4**[*]	67.7 ± 15.2	**139.6 ± 40.0**[*]	**0.66 ± 3.07**[*]
Alcohol consumption										
No	27.9 ± 3.4	113.1± 10.6	68.4 ± 8.3	67.2 ± 10.3	8.67 ± 1.85	1.42 ± 1.95	170.7 ± 1.6	65.5 ± 14.2	129.3 ± 34.5	-0.63 ± 3.22
Yes	27.1 ± 3.2	**117.4 ± 12.8**[*]	71.6 ± 8.8	68.5 ± 9.0	8.08 ± 1.68	1.36 ± 1.70	165.7 ± 1.5	65.0 ± 15.2	**145.6 ± 42.1**[*]	0.52 ± 2.60
PA (METs-min/week)										
T1	27.8 ± 3.1	112.7 ± 11.0	69.7 ± 8.5	67.4 ± 9.5	8.08 ± 1.75	1.34 ± 1.80	174.2 ± 1.7	64.5 ± 15.8	133.5 ± 40.4	0.13 ± 2.86
T2	28.2 ± 3.3	114.9 ± 11.6	68.8 ± 8.7	68.4 ± 8.5	8.67 ± 1.82	1.45 ± 1.90	165.7 ± 1.6	66.3 ± 13.7	130.9 ± 32.9	0.23 ± 3.15
T3	26.8 ± 3.4	112.7 ± 9.9	67.7 ± 8.7	65.1 ± 13.0	9.11 ± 1.91	1.42 ± 2.12	172.4 ± 1.5	65.9 ± 12.7	128.9 ± 35.3	-0.63 ± 3.34

rMedDiet score (point)										
T1	27.6 ± 3.1	113.8 ± 11.5	68.5 ± 8.7	68.5 ± 9.0	8.84 ± 1.76	1.49 ± 1.84	165.7 ± 1.6	63.1 ± 12.3	134.7 ± 36.7	0.33 ± 2.84
T2	27.6 ± 3.3	112.7 ± 10.9	68.9 ± 8.3	66.4 ± 11.5	8.49 ± 1.80	1.38 ± 1.92	170.7 ± 1.6	67.3 ± 14.7	129.1 ± 35.3	-0.42 ± 3.23
T3	28.3 ± 3.8	115.3 ± 10.5	69.5 ± 8.5	67.7 ± 8.7	8.24 ± 1.97	1.36 ± 2.08	177.7 ± 1.6	65.3 ± 16.0	131.7 ± 36.8	0.38 ± 3.36

Values are expressed in means ± SD (standard deviation). Abbreviations: BMI, body mass index; GWG, gestational weight gain; IOM, Institute of Medicine; PA, Physical Activity; METs, metabolic equivalents; T, tertile; rMedDiet, Mediterranean diet; SBP, systolic blood pressure; DBP, diastolic blood pressure; HOMA-IR, Homeostatic Model Assessment for Insulin Resistance; HDL-c, high-density lipoprotein-cholesterol; LDL-c, low-density lipoprotein-cholesterol; CCR, clustered cardiometabolic risk. The significance of the numbers in bold is p-value < 0.05. * $p<0.05$ compared with the first category as derived from ANOVA or Student's T-test, as appropriate. **The significance of the numbers in bold is p-value < 0.001. ¶ Recommendations for GWG according to IOM guidelines are: initial BMI <18.5 kg/m^2, total weight gain 12.5–18 kg; BMI 18.5–24.9 kg/m^2, total weight gain 11.5–16 kg; BMI 25.0–29.9 kg/m^2, total weight gain 7–11.5 kg; and BMI ≥30 kg/m^2, total weight gain 5–9 kg. †In the first trimester of pregnancy (except for GWG). ‡Geometric means of log-transformed values. §A higher clustered cardiometabolic status signifies higher cardiometabolic risk.
